# Static MLC transmission simulation using two‐dimensional ray tracing

**DOI:** 10.1002/acm2.13646

**Published:** 2022-05-20

**Authors:** David P. Adam, Bryan P. Bednarz, Sean P. Frigo

**Affiliations:** ^1^ Department of Medical Physics University of Wisconsin‐Madison School of Medicine and Public Health Madison Wisconsin USA; ^2^ Department of Human Oncology University of Wisconsin‐Madison School of Medicine and Public Health Madison Wisconsin USA

**Keywords:** HDMLC calibration, MLC modeling, ray tracing

## Abstract

**Purpose:**

We investigated the hypothesis that the transmission function of rounded end linearly traveling multileaf collimators (MLCs) is constant with position. This assumption is made by some MLC models used in clinical treatment planning systems (TPSs) and in the Varian MLC calibration convention. If not constant, this would have implications for treatment plan QA results.

**Methods:**

A two‐dimensional ray‐tracing tool to generate transmission curves as a function of leaf position was created and validated. The curves for clinically available leaf tip positions (−20 to 20 cm) were analyzed to determine the location of the beam edge (half‐attenuation X‐ray [XR]) location, the beam edge broadening (BEB, 80%–20% width), as well as the leaf tip zone width. More generalized scenarios were then simulated to elucidate trends as a function of leaf tip radius.

**Results:**

In the analysis of the Varian high‐definition MLC, two regions were identified: a quasi‐static inner region centered about central axis (CAX), and an outer one, in which large deviations were observed. A phenomenon was identified where the half‐attenuation ray position, relative to that of the tip or tangential ray, increases dramatically at definitive points from CAX. Similar behavior is seen for BEB. An analysis shows that as the leaf radius parameter value is made smaller, the size of the quasi‐static region is greater (and vice versa).

**Conclusion:**

The MLC transmission curve properties determined by this study have implications both for MLC position calibrations and modeling within TPSs. Two‐dimensional ray tracing can be utilized to identify where simple behaviors hold, and where they deviate. These results can help clinical physicists engage with vendors to improve MLC models, subsequent fluence calculations, and hence dose calculation accuracy.

## 1 INTRODUCTION

The majority of linac‐based photon treatment delivery systems (TDSs) in use are of a c‐arm design, and most have a multileaf collimator (MLC) aperture whose leaves travel linearly and have ends that are rounded. Consequently, there is an extensive body of literature on the mechanical and dosimetric properties of rounded end linearly traveling MLCs^1^, ^2^, ^3^, ^4^, ^5^, ^6^, ^7^, ^8^ (RELT‐MLCs).

Along with requiring less space in the TDS head, RELT‐MLCs are designed to have a nearly constant beam edge broadening (BEB, 80%–20% width) across all field sizes. In addition, the XR beam edge position, as demarcated by the 50% attenuation level, has been found to be approximately constant relative to the light field edge, deviating on the order of tenths of a millimeter.^9^ This allows calibrating MLC positions to the visible light field edge, which is the Varian convention.

These benefits come at an expense. The first is the need for a positioning table with the TDS, in order to place the visible light field edge at the calibrated position. The second is that the treatment planning system (TPS) needs to know of the calibration convention and handle leaf positioning accordingly.^1^ Third, RELT‐MLCs have additional BEB due to the rounded ends. In contrast, divergently matched MLCs, the ends of which are flat surfaces that follow the beam direction at the tip position,^10^ do not suffer from either position calibration challenges or BEB effects.

Owing to their prevalence, the RELT‐MLC transmission has been the subject of many investigations. Lorenz, for example, established that the transmission behavior is not constant for all beam sizes.^11^ Kumaraswamy experimentally demonstrated intraleaf body transmission spatial variation in the horizontal plane.^12^ Chang measured and showed that the transmission changes as a function of depth in water.^13^ The RELT‐MLC transmission is actually spatially and spectrally variant and not constant as assumed in many TPSs.

The transmission behavior has been modeled multiple ways with varying degrees of approximations. These include analytical solutions,^9^ ray‐tracing,^1^, ^3^, ^6^ and Monte Carlo methods.^6^ Although there has been a fair amount of work investigating the spatial dependence of MLC transmission, there has yet to be an exhaustive computational characterization of transmission curves at all clinically available leaf positions. In contrast to the wide range of model studies, the MLC models used in clinical TPSs often employ a simplified representation of the transmission function.

For example, the Eclipse model assigns a zero height and a constant leaf body transmission value.^14^ A region at the tip is given a transmission value of unity, which effectively applies a shift in leaf position within the TPS prior to calculation. The offset parameter value is often chosen to match the calculated and measured integrated fluence at a point on central axis (CAX) as determined with beams with variable width‐sweeping gaps.^6^ This is manifested in the Eclipse dosimetric leaf gap (DLG) parameter. The DLG value and the transmission are constant with leaf position in Eclipse.

In RayStation, the MLC model is slightly more complex. In addition to shifting the leaf position internally in what is termed the “calibration” region, and assigning a unit transmission value over that shift, a second region thereafter is added called the “leaf tip width.” In the latter, the transmission value is the square root of the MLC body transmission value.^15^ In all the regions, the assigned transmission values are constant. Aside from the RayStation calibration region width, which can vary as a polynomial with position, there are no other possible transmission variations with leaf position.

In these treatments, central to the position calibration and BEB of RELT‐MLC beams is that of constancy. First, the offset between visible ray (VR) and 50% XR edge is treated as constant with leaf position. Second, the BEB is also considered constant with position. Both assumptions are present in the TrueBeam TDS and Eclipse TPS and are largely present in the RayStation TPS. However, if these assumptions do not hold, calculation differences may be exacerbated for small targets versus large ones or those lying far off‐axis. For example, Vial showed that TPS MLC model parameters were quite sensitive to slight changes and small tweaks to these parameter values could improve the dosimetry at large off‐axis positions.^16^

In this work, we created a tool that investigates the spatial dependence of RELT‐MLC transmission. This is done using two‐dimensional (2D) ray tracing in the direction of leaf travel through the CAX for a single leaf. By analyzing transmission curves for different MLC positions, we characterize the beam edge position and BEB for a commercial MLC, the Varian high‐definition MLC (HDMLC). We discovered that these properties can be treated as constant up to a certain break point, but thereafter, cannot. This divides the leaf position space into essentially two distinct zones. We then extended the result to determine zone boundaries as a function of leaf radius. This work illustrates that a TPS model that assumes constancy may be valid near CAX but potentially may not meet accuracy goals outside the constancy zone. These results can help clinical physicists engage with TPS vendors to improve MLC models, subsequent fluence calculations, and hence dose calculation accuracy.

## 2 MATERIALS AND METHODS

We summarize the materials and methods in the following section. The abbreviations used in this work are summarized in Table 1.

**TABLE 1:** Table of abbreviations

Acronym	Meaning
BEB	Beam edge broadening
BR	Boundary‐ray
CAX	Central axis
DLG	Dosimetric leaf gap
HDMLC	High‐definition MLC
MLC	Multileaf collimator
RELT	Rounded end linearly traveling
SCD	Source–collimator distance
SSD	Source surface distance
TDS	Treatment delivery system
TPS	Treatment planning system
TR	Tip ray
TZW	Tip zone width
VR	Visible ray
*X_break_*	Location where property begins to deviate
XR	(50% attenuation) X‐ray
XVO	X‐ray visible offset (XR‐VR)

### 2.1 Leaf model

A geometry representative of a single‐MLC leaf symmetric about its midline was represented in 2D as a rectangle and a fitted circle to generate a rounded end at the tip. There are three input parameters to the MLC model: height, radius, and transmission.

A height of 6.60 cm was calculated using the vendor‐supplied drawings.^17^, ^18^ Determining a single representative value is complicated by the grooved guides on the top and bottom of the HDMLC, as well as having a drive screw clearance slot within the body. Both types of features lead to missing tungsten material. The screw slot begins about 2 cm from the tip and was neglected. The guides, however, extend to the tips and end at a chamfer. This added material was considered in determining the leaf height parameter value by weighting the guide heights by the fraction of space filled by materials.

A radius of 16.00 cm for the fitted circle was obtained from vendor‐supplied drawings.^17^, ^18^ It was found that a single circle represented the entire tip region well.

The leaf transmission on CAX ($T_{⊥}$) was determined from the average of measured literature values,^13^, ^19^, ^20^ using a Farmer chamber at an source surface distance of 90.0 cm at a depth of 10.0 cm.^13^, ^20^, ^21^ For a flattened 6‐MV beam (denoted as X06F), the transmission value used in the simulations was 1.22%. This transmission value corresponds to a linear attenuation coefficient (μ) of 0.668 cm^−1^ and a leaf height (*h*) of 6.60 cm, assuming simple exponential attenuation from the relation given in the following equation:

(1) $$\mu \;=\;\frac{-1}{h}\ln\left(T_{⊥}\right)$$

The beam energy spectrum was represented by the single transmission value, and no energy‐dependent treatment was performed.

The specific TrueBeam HDMLC machine geometry was also taken from vendor‐supplied drawings. This included a source–collimator distance of 51.0 cm, and a source–axis distance of 100.00 cm. The TrueBeam source is less than 1 mm in size and was represented as a point.^22^ As a result, we report all edge widths as BEB and not penumbra, as the former is due to the MLC end alone, whereas the latter only applies to a source of finite size and divergently matched aperture.

#### 2.1.1 Ray classification

**FIGURE 1:**
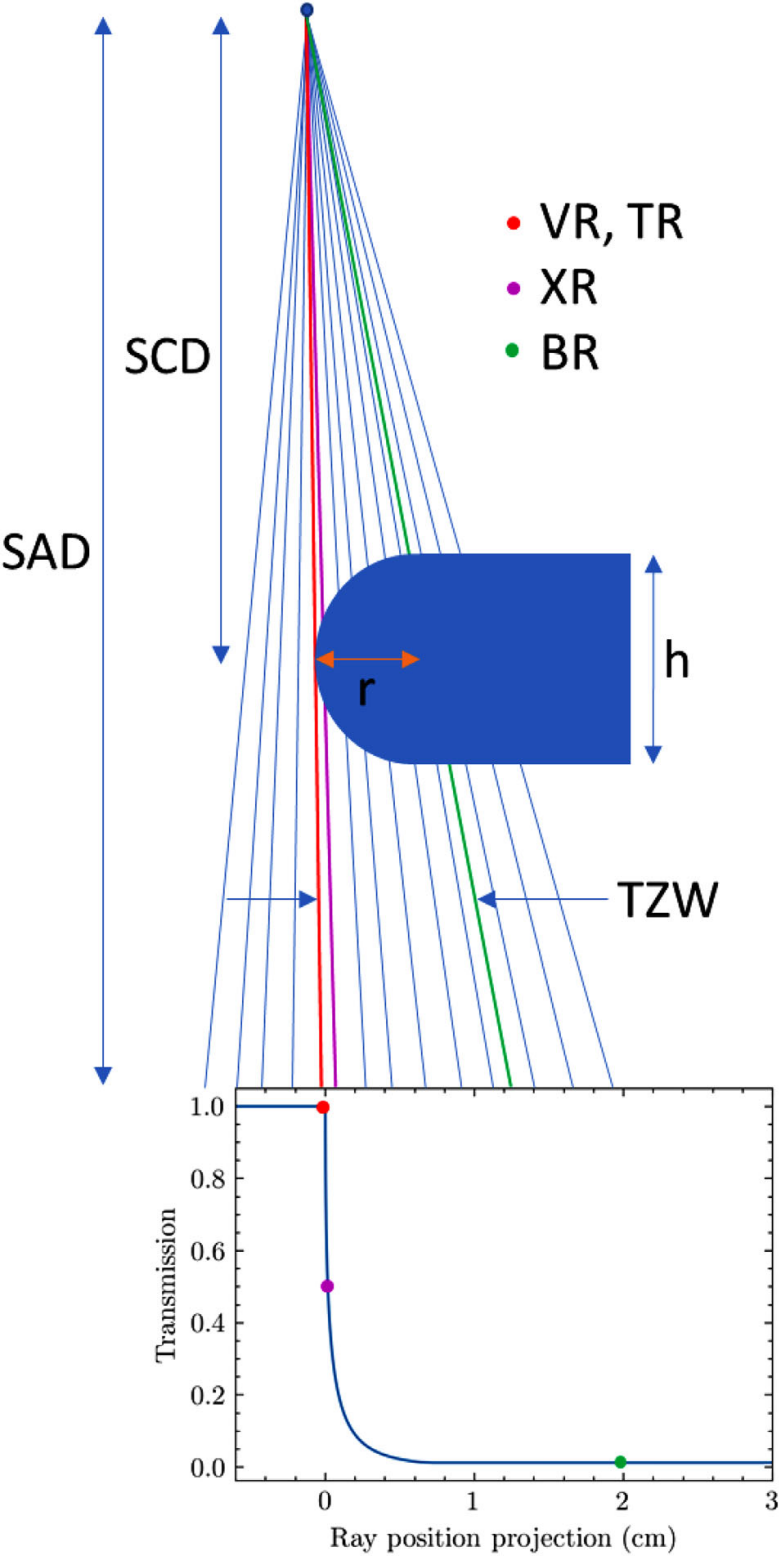
Diagram depicting geometry, ray classification, and relation to a transmission curve. The diagram is not to scale, and the MLC features are exaggerated. For clarity, the leaf is on the CAX, and thus the TR and VR are at the same location. CAX, central axis; MLC, multileaf collimator; TR, tip ray; VR, visible ray

We use nomenclature similar to that described by Vial^16^ in describing different MLC ray types. They are depicted in Figure 1. The tip‐ray (TR) refers to the position of the distal end of the leaf relative to the collimator CAX. The VR refers to the position of a ray tangential to the rounded leaf end. The XR refers to the position of the ray that is attenuated by half, that is, the one which defines the beam edge at 50% intensity relative to the open beam.^16^ The boundary‐ray (BR) refers to the first ray from the tip that intersects only the rectangular portion of the leaf, that is, the ray enters the body at the top and exits the body at the bottom. It is the boundary where rays have a nonzero portion of their path length in the tip. In the figure, the leaf is centered on the CAX for clarity, and thus the TR and VR are equivalent. All positions in this work are projected to the isocenter plane. Also, we assume a flat incident fluence profile to focus on MLC‐specific effects only.

#### 2.1.2 Ray tracing

The ray‐tracing tool was developed using Python with the Pyrr^23^ package. Rays cast from a point source to the isocenter plane are swept from negative to positive *x*‐coordinates in increments of 0.00001 cm. The region of interest is across the length of the parked MLC's geometrical representation from the end of the tip to the first path traversing only the rectangular portion of the leaf. The length of the intersecting chord (*r*) is recorded for each cast ray, and the linear attenuation coefficient (*μ*) is applied to determine the transmission (*T*(*r*)) of that ray according to the following equation:

(2) $$T\left(r\right)\;=\;e^{-\mu r}$$

The VR is determined from the last swept ray position in which the transmission is unity, that is, the tangent, and the XR is determined from where the swept ray transmission is 50% of unity. The ray tracing conducted is effectively a two‐dimensional simulation on a single leaf of finite height and infinite width crossing the beam CAX, that is, it is one of a pair that are assumed to be the central most leaf pair in an MLC bank.

### 2.2 Transmission curves

#### 2.2.1 Generation

Transmission curves were generated by ray tracing using the sweeping ray algorithm for different fixed MLC leaf tip positions. The leaf tip *x*‐coordinate was stepped in 1‐cm increments to model the leaf translating horizontally in the one‐dimensional plane of the leaf body. The transmission was calculated as the exponential attenuation of the determined ray path length per Equation (2). By repeating for leaf positions of −20 to +20 cm, we were able to generate transmission curves as a function of leaf off‐axis position across all clinically available leaf positions.

#### 2.2.2 Validation

The ray‐tracing algorithm was validated by reproducing the results published by Boyer,^9^ where we used their dimensions (8.00‐cm tip radius, a height of 6.13 cm, and a half value layer of 0.950 cm) as ray‐tracing tool parameter values. The resultant transmission curves were used to generate the XR–VR offset (XVO) as a function of tip position, which was compared to the results from analytical functions published by Boyer.^9^ Further validation was performed by identifying the behavior of intersection locations at edge cases and analyzing those graphically. This was to ensure that results were not affected by interpolation or sampling artifacts.

#### 2.2.3 Analysis

Using the VR from the tangent of the circular tip, the generated transmission curves as a function of TR were analyzed. Using the locations of the XR and BR, the associated XVO was determined. Additionally, the BEB was calculated from the difference in position between the 80% and 20% points of full transmission on each curve. The variations in XVO and BEB were plotted against TR position, and in cases where the plotted values deviated from near constancy, the TR value was recorded and denoted as *X_break_*.

### 2.3 Geometrical analysis

#### 2.3.1 Intersection

The intersection locations of a ray with the tip and body were determined by ray‐box (leaf body) and ray‐cylinder (leaf tip) intersections. Routines were used to determine intersection locations, and from these necessary path lengths and attenuations were directly calculated in both the leaf body and leaf tip. This allowed us to decompose a ray into a segment lying within the tip and a segment lying within the body, that is, to perform a path length analysis.

#### 2.3.2 Tip zone width (TZW)

The BR–VR offset, also referred to as the tip zone width (TZW), measures the extent of the contribution of the tip zone to the transmission curve. In other words, it is the projection of the region where rays enter and/or exit the rounded leaf end portion of an MLC leaf. This was calculated from the determined BR and VR positions.

## 3 RESULTS

The results for the tool validation, generated transmission curves, and TZW as a function of tip position are presented later. Conditions where the XR position deviates greatly from the VR's, and the BEB increases significantly, were identified and explored further as a function of leaf tip radius.

### 3.1 Tool validation

**FIGURE 2:**
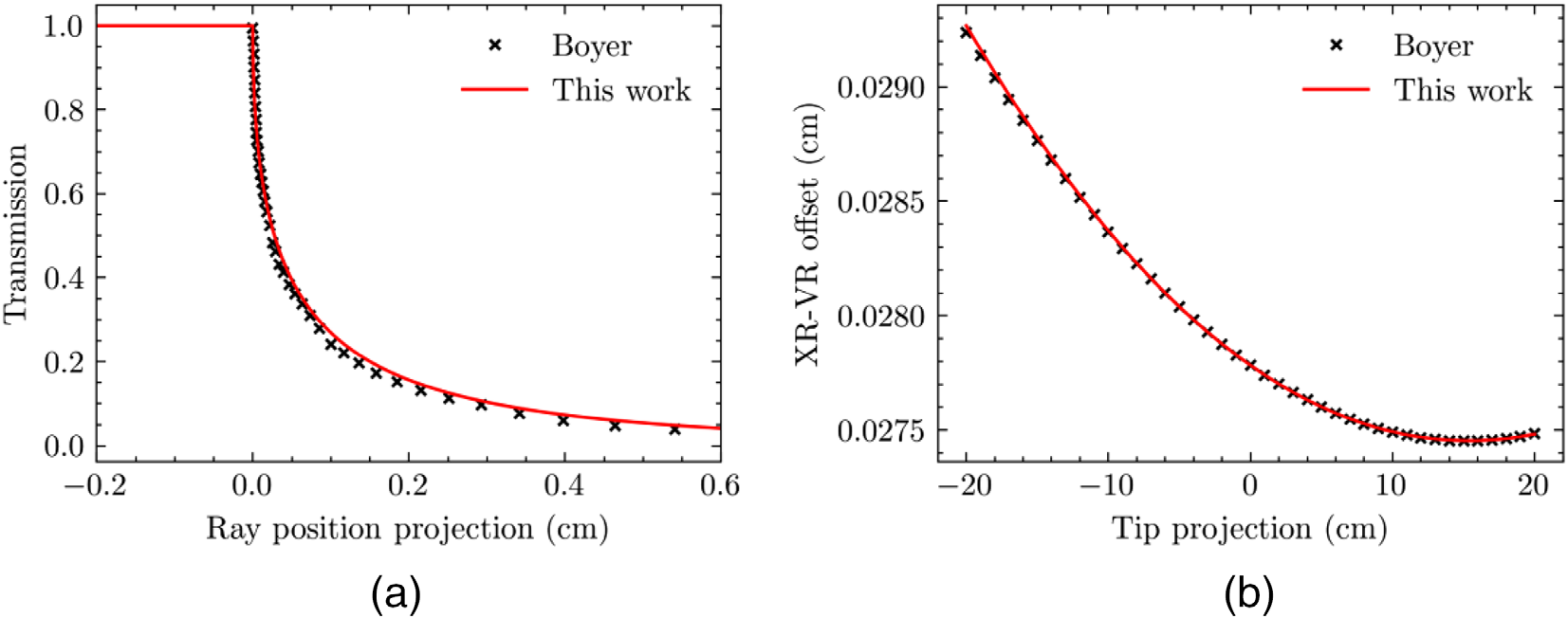
Comparison of (a) transmission curves and (b) XVO between the ray‐tracing tool and the analytical expressions published by Boyer.^9^ XVO, X‐ray visible offset

A comparison between the ray‐tracing tool generated and the Boyer transmission curves for a TR of 0.00 cm (on CAX) is shown in Figure 2a. A comparison of the XVO produced using ray tracing to the analytical functions given by Boyer is shown in Figure 2b. The differences between the transmission curves are ascribed in part to uncertainty in the manual data extraction process from the publication figure. The maximum difference between the XVO curves is less than 0.1%.

### 3.2 Transmission curves

**FIGURE 3:**
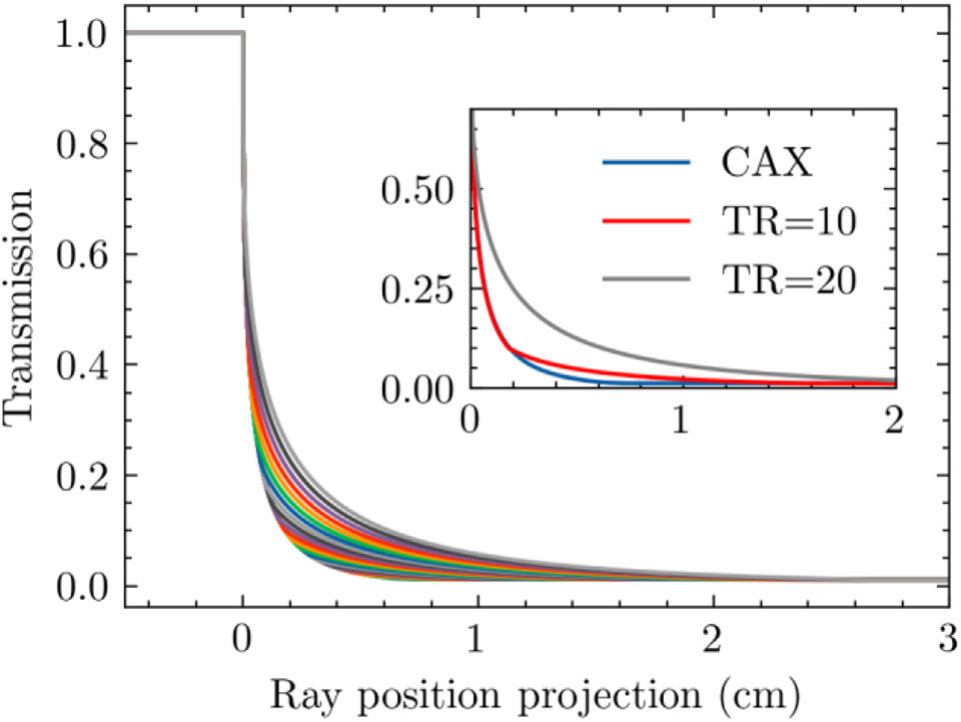
Transmission curves at different TR values. The inset figure includes only TR values of 0 (CAX), 10, and 20 cm to show the spread. Other positions lie in a progression from one curve to the other. CAX, central axis; TR, tip‐ray

Generated transmission curves for differing TR positions, relative to each position's VR, are given in Figure 3. The inset shows the transmission curves that differ the most, those at a TR of 0, 10, and 20 cm. We mostly see a progression in the bend region, where the transmission increases as the leaf moves distally from CAX. For greatest TR values, the transmission begins to deviate significantly (Figure 3 inset). The curve with the tip at CAX decreases fastest with ray position, whereas for larger TR values, the transmission decreases more slowly.

### 3.3 Geometrical analysis

We present results detailing the sweeping ray algorithm that calculates the ray lengths in each portion of the leaf and the corresponding VR and XR intersections.

#### 3.3.1 Intersection

**FIGURE 4:**
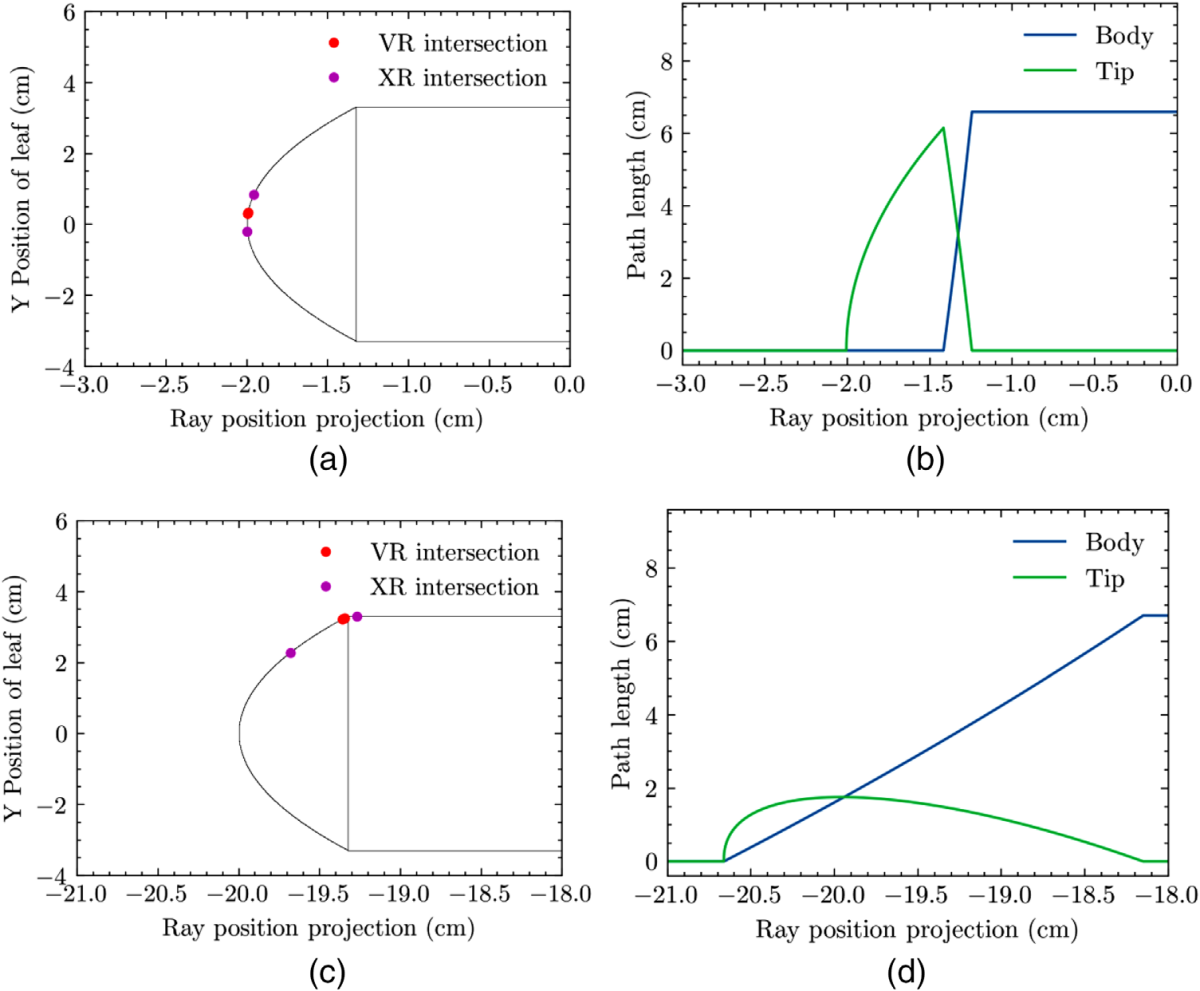
Depiction of leaf intersections for an HDMLC leaf tip model at a TR of (a) −2 and (c) −20 cm. Leaf boundaries are depicted in black, the VR is depicted as a red point, and the XR intersection points are portrayed in magenta. A path length analysis is shown in (b) and (d), with the corresponding ray lengths in the tip (green) and body portions of the leaf (blue). HDMLC, high definition‐multileaf collimator; TR, tip‐ray; VR, visible ray; XR, X‐ray

Figure 4 details the intersection points and ray lengths generated by the ray‐tracing algorithm for the HDMLC at TR values of −2 and −20 cm. The leaf is fixed and rays are swept across the leaf model to calculate the XR and VR intersection points, as well as the length of each swept ray in the two distinct regions of the leaf. For a TR of −2 cm, the XR and VR intersections occur on the rounded tip end, whereas at −20 cm, the VR is at the circle‐body transition. In the latter case, the XR intersects the body when entering, and the rounded tip when exiting.

For a TR of −2 cm, the rays mainly pass through the tip, and there is a small region in which rays pass through both the body and the tip. The rays intersecting the tip occur in a region projecting less than 0.8‐cm wide in the isocenter plane, and the maximum ray length within the tip exceeds 6 cm. Alternatively, for a TR of −20 cm, almost all rays pass through both the body and the tip. The rays intersecting the tip project over a much wider distance in the isocenter plane, approximately 2.5 cm. The maximum ray length in the tip, in contrast to the −2 cm position, does not exceed 2 cm, and we see that the paths are always a mixture of tip and body segments, whereas for positions closer to the CAX, most ray paths lie purely within the tip region until transitioning to a mixture as the rays approach the body, that is, closer to the BR.

#### 3.3.2 Tip zone width (TZW)

**FIGURE 5:**
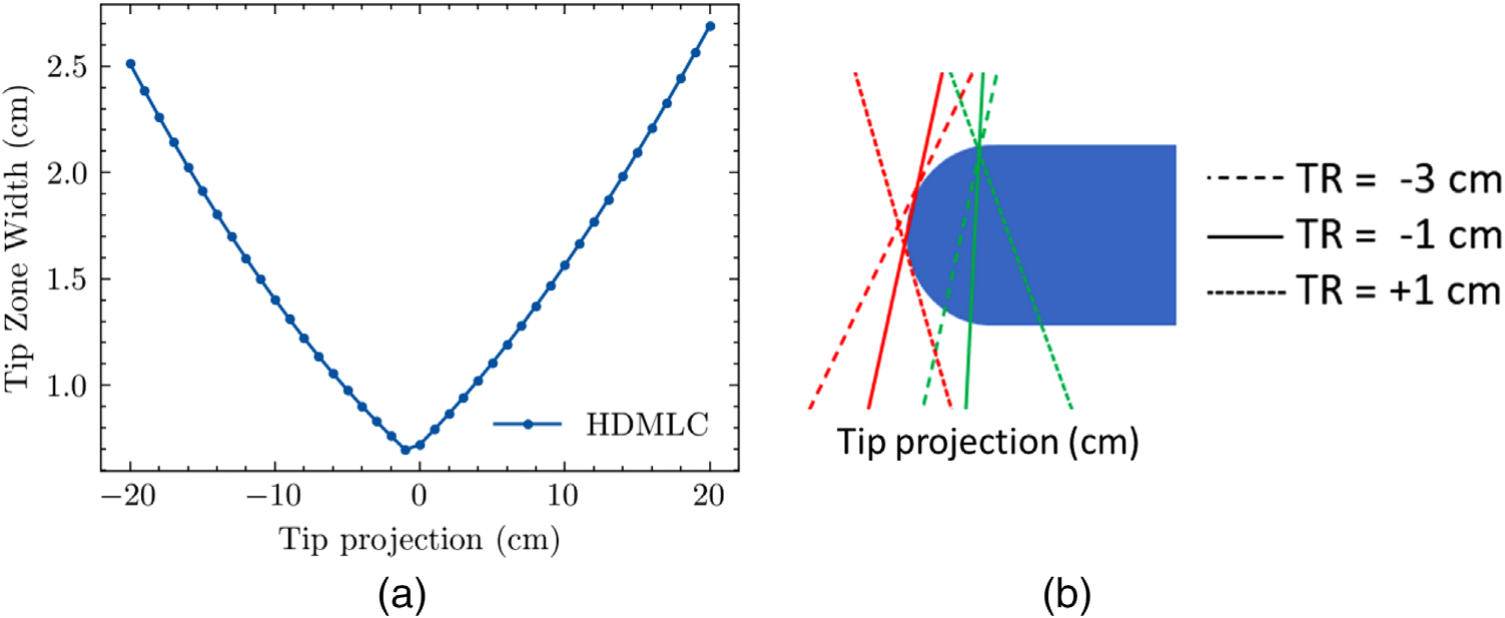
(a) TZW for the HDMLC as a function of TR and (b) diagram depicting the geometric rationale as to why the TZW is minimized at a TR of −1 cm. In (b), the diagram is not to scale. HDMLC, high‐definition multileaf collimator; TR, tip‐ray; TZW, tip zone width

Figure 5a depicts the calculated TZWs generated as a function of off‐axis position for the HDMLC moving from right to left, that is, from positive to negative *x*‐coordinate. The TZW is solely determined by geometry and has no dependence on energy spectrum or transmission. The HDMLC TZW has minimum at a TR of −1 cm and a maximum at 20 cm. The minimum TZW occurs at −1 cm because the length of the BR is minimized at this position, that is, the angle subtending the BR and VR becomes minimized. Figure 5b depicts this graphically. The TZW increases proportionally from the minimum as the width of the angle subtending the tip increases for TR values further away from CAX. In other words, the curve in Figure 5a would have a minimum about *x* = 0 if plotted against the BR position.

### 3.4 Constancy break

#### 3.4.1 XR–VR offset (XVO)

**FIGURE 6:**
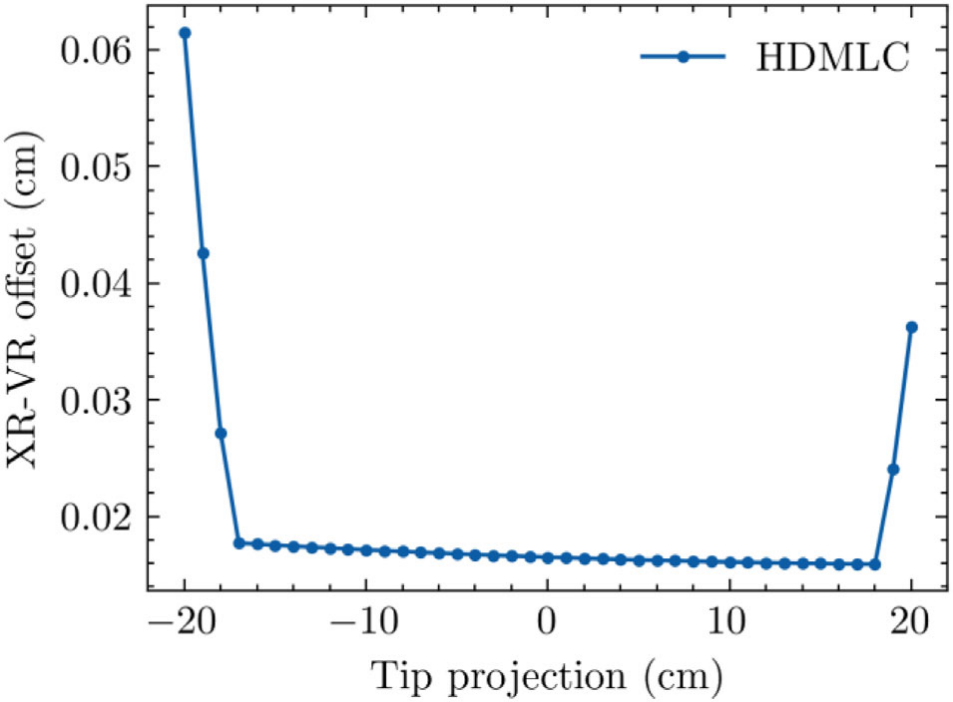
XVO as a function of tip position for the HDMLC as a function of TR. HDMLC, high‐definition multileaf collimator; TR, tip‐ray; XVO, X‐ray visible offset

An interesting geometric phenomenon was observed for the HDMLC in which the XVO breaks from quasi‐static behavior. As depicted in Figure 3, the transmission curves broaden as the leaf is moved further off‐axis, and qualitatively the difference between the XR and VR becomes greater. Figure 6 depicts the XVO for the HDMLC quantitatively as a function of the TR, showing deviations at a TR < −17 and TR > 18 cm. We denote the location where this occurs as *X_break_*.

To confirm the validity of this phenomenon, the intersection points of the ray‐tracing algorithm were plotted alongside the calculated XR and VR intersections for a TR of −2 cm and a TR of −20 cm as is depicted in Figure 4. In the case where the TR is −2 cm (Figure 4a), and within the quasi‐static XVO region as depicted in Figure 6, both the XR and VR intersection points occur on the rounded leaf tip. In contrast, when the TR is −20 cm (Figure 4c) and outside of the quasi‐static XVO region, the XR and VR intersections move to the body portion of the leaf. The *X_break_* behavior is attributable to when the intersection points move off of the rounded leaf end and onto the leaf body.

**FIGURE 7:**
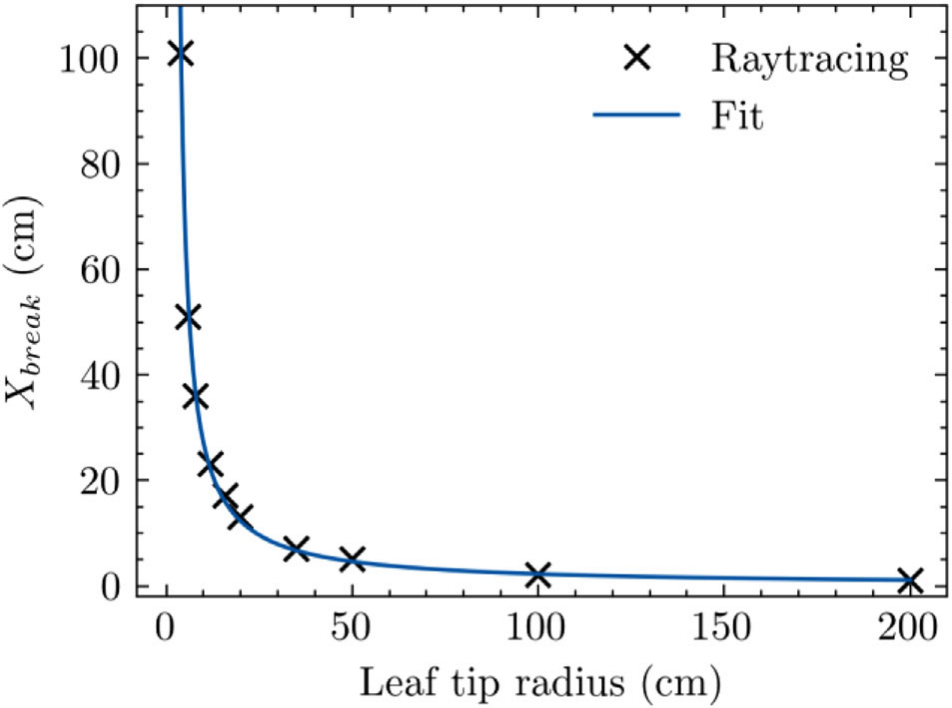
Depiction of TR at which the XVO *X_break_* occurs as a function of tip radius. The smooth line is a fit to the calculated data points. TR, tip‐ray; XVO, X‐ray visible offset

To extend this observed phenomenon into a more generalized form, Figure 7 depicts the computed TR in which the XVO *X_break_* occurs as a function of the leaf tip radius. It shows that as the leaf tip radius becomes larger, the TR at which *X_break_* occurs becomes smaller, that is, closer to CAX. For leaves with small radii (<4 cm), the TR at which *X_break_* occurs lies in excess of 100 cm from CAX. In practical terms, this shows that as the radius is larger, the TR at which the *X_break_* occurs happens sooner, that is, the zone of constancy shrinks with leaf radius.

#### 3.4.2 Beam edge broadening (BEB)

**FIGURE 8:**
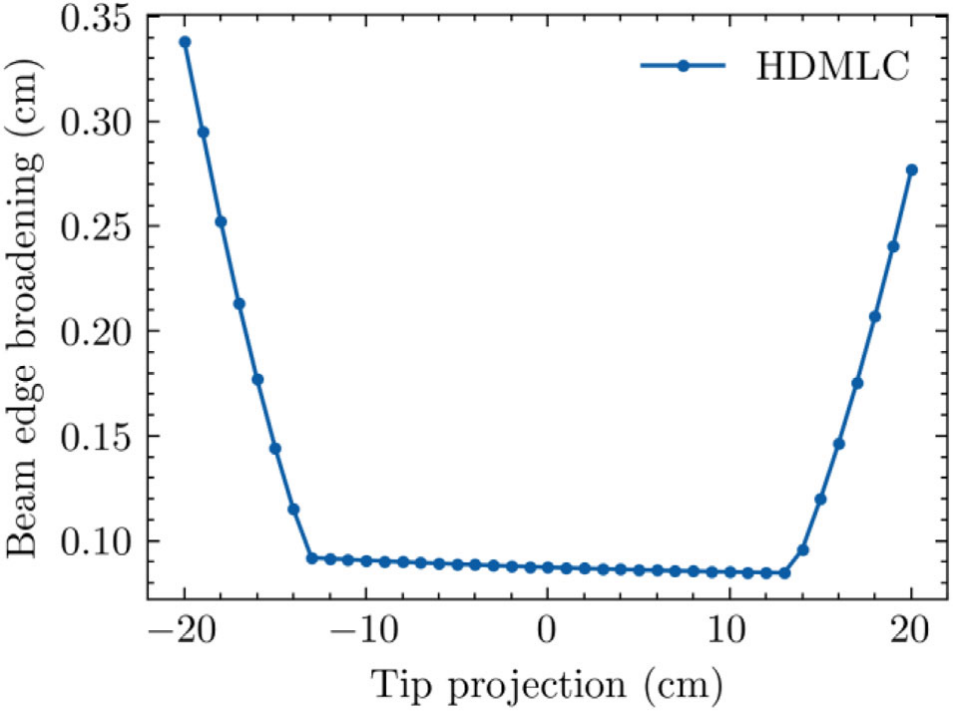
BEB (80%–20% width) as a function of tip position for HDMLC. BEB, beam edge broadening; HDMLC, high‐definition multileaf collimator

Similar behavior was observed for the BEB derived from the calculated transmission curves, as shown in Figure 8. The HDMLC BEB as a function of TR in a central region is essentially constant but then deviates significantly in a similar manner as the XVO (Figure 6). Here, the BEB *X_break_* occurred sooner, at TR < −13 cm and TR > 13 cm.

**FIGURE 9:**
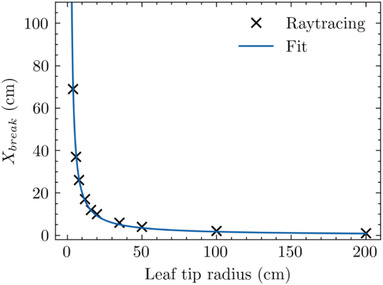
Depiction of TR at which the BEB *X_break_* occurs as a function of tip radius. The smooth line is a fit to the calculated data points. BEB, beam edge broadening; TR, tip‐ray

Figure 9 depicts the computed TR in which the BEB is a function of the leaf tip radius. Similar to that of the XVO *X_break_*, it shows that as the leaf tip radius becomes larger, the BEB *X_break_* occurs closer to CAX.

## 4 DISCUSSION

The overarching aim of this work was to generate transmission curves that can be used to predict and inform static RELT‐MLC behavior. The results shed light on physical phenomena that need to be considered by a TPS model. Studies of the HDMLC demonstrated variation in transmission curves with position, and derived parameters across these positions showed that the assumption of constancy does not hold outside a central zone about the CAX.

First, we reproduced previously published results using the leaf geometry in the work by Boyer.^9^ The ray tracing for both the transmission curves at a tip position on CAX, as well as the XVO for all tip positions, agreed well with the analytical approach to within data extraction uncertainties.

Second, our results demonstrate that there is an effective region in which quasi‐static behavior is applicable for the HDMLC, and a region in which the behavior is divergent. Our finding of *X_break_*, in which both the XVO and BEB deviate from CAX values, was shown to be a real phenomenon, as shown by the intersection analysis. For the HDMLC, the XR intersections for off‐axis positions occurred within the leaf body, leading to a larger difference in the XVO, than for MLC positions closer to the CAX. We note that although not exactly the same, according to our model, the Varian Millennium MLC, with an 8.0‐cm tip radius, would have its *X_break_* approximately around 30 cm per Figures 7 and 9, outside clinically available positions.

We find from Figures 6 and 7 that the Varian VR calibration convention is more accurate for leaf positions closer to CAX and as the leaf radius decreases. Similarly, from Figure 8, the BEB is more consistent for values closer to CAX. For the divergent region, a calibration based on the XR, as is the Elekta convention, is warranted; however, this still would not address the BEB behavior.

Theoretically, *X_break_* could have dosimetric consequences for beam boundaries located significantly off‐axis. Based on the HDMLC results, we estimate that static beam edge locations and widths could be different by up to 0.1 and 0.3 cm, respectively. A clinical scenario where this would be impactful is in the treatment of small targets off‐axis, for example, multiple metastases using stereotactic radiosurgery methods. However, under most static beam circumstances, the constancy assumption holds up quite well for both the HDMLC and MMLC.

These geometric phenomena could have clinically relevant consequences for treatment plans, examples of which have been shown previously.^24^ For the HDMLC, the results for the XVO imply that lateral targets may be more prone to QA failure than centrally located targets. Due to potential parameter value sensitivities, dosimetric effects may be exacerbated for small targets and/or single‐isocenter multi‐metastasis target definitions spanning a large treatment region.

The 2D simulation was conducted as to tie it to the simplistic MLC models in widely used TPSs. With this approach, the number of confounding factors was minimized. For example, it allowed us to employ a single measured transmission value. It was also easier and more expedient than more complicated modeling approaches. The effect of source size and spectrum was briefly considered but were found to only contribute second‐order effects. A 3D ray‐tracing approach was explored, but the benefit was determined not worth the computational complexity arising from using a polyenergetic and spatially varying transmission function in 2D. Monte Carlo methods were not pursued, as they are much more inflexible and would have required extensive characterization of the source.

In this light, there are inherent limitations of the 2D ray‐tracing tool used in this work. One deficiency is that there is no *z* or *y* spatial dependence of the transmission; the single value we used only represents the value at one depth, whereas there is literature suggesting that there is a lateral^12^ and depth dependence.^13^ Higher dimensional simulations may be more revealing of the spatial dependence of the transmission, which could also play a role in the eventual 3D TERMA distribution in dose calculations. Additionally, tongue and groove effects were not considered, and the exact density and composition of the leaves are also approximated. By using measurements with a large ion chamber, we take into account contributions of inter‐ and intraleaf leakage and scatter to the downstream fluence.

## 5 CONCLUSIONS

We have developed a 2D ray‐tracing tool to generate and analyze RELT‐MLC transmission curves for a c‐arm TDS. We have validated the tool against prior work. From these curves, we have demonstrated how the curves vary with position as well as shed light on the variation of static beam edges with leaf position for the Varian HDMLC. We identify a geometric zone of constancy, wherein the XVO and BEB can be treated as constant. However, outside of this zone, significant deviations occur that can affect calculating the fluence and hence the dose distribution. These limitations need to be considered when calibrating RELT‐MLCs to the light field and also when modeling within TPSs.

Future work should seek to extend this study to analyze dynamic RELT‐MLCs, to more fully address the following questions. Are current MLC models in Eclipse and RayStation sufficient? Is it possible that divergently matched MLCs offer superior performance in comparison to horizontally traversing rounded end MLCs? What can be learned by a systematic ray‐tracing study of dynamic single and paired leaves? Ultimately, we believe that a fully ray‐traced transmission function will more accurately characterize the radiation beam such that both small and large targets as well as static and dynamic deliveries are modeled more accurately.

## CONFLICT OF INTEREST

BB is a cofounder and has an ownership interest in Voximetry, Inc., a nuclear medicine dosimetry company in Madison, WI.

## AUTHOR CONTRIBUTION

All listed authors earlier contributed equally to the intellectual content of the manuscript, including the design, acquisition, analysis, and interpretation. They participated equally in drafting, revising, and interpreting the material. All authors have read and approved the final submitted version of the manuscript. Additionally, none of the authors have any outside funding sources that contributed to this work and have not published the data or results prior.
